# Antitumor activity of a potent MEK inhibitor, TAK-733, against colorectal cancer cell lines and patient derived xenografts

**DOI:** 10.18632/oncotarget.5949

**Published:** 2015-10-01

**Authors:** Christopher H. Lieu, Peter J. Klauck, Patrick K. Henthorn, John J. Tentler, Aik-Choon Tan, Anna Spreafico, Heather M. Selby, Blair C. Britt, Stacey M. Bagby, John J. Arcaroli, Wells A. Messersmith, Todd M. Pitts, S. Gail Eckhardt

**Affiliations:** ^1^ Department of Medicine, Division of Medical Oncology, University of Colorado Anschutz Medical Campus, Aurora, CO, USA

**Keywords:** MEK, colorectal cancer, patient derived xenografts, TAK-733

## Abstract

**Background:**

CRC is a significant cause of cancer mortality, and new therapies are needed for patients with advanced disease. TAK-733 is a highly potent and selective investigational novel MEK allosteric site inhibitor.

**Materials and Methods:**

In a preclinical study of TAK-733, a panel of CRC cell lines were exposed to varying concentrations of the agent for 72 hours followed by a sulforhodamine B assay. Twenty patient-derived colorectal cancer xenografts were then treated with TAK-733 *in vivo*. Tumor growth inhibition index (TGII) was assessed to evaluate the sensitivity of the CRC explants to TAK-733 while linear regression was utilized to investigate the predictive effects of genotype on the TGII of explants.

**Results:**

Fifty-four CRC cell lines were exposed to TAK-733, while 42 cell lines were deemed sensitive across a broad range of mutations. Eighty-two percent of the cell lines within the sensitive subset were BRAF or KRAS/NRAS mutant, whereas 80% of the cell lines within the sensitive subset were PIK3CA WT. Twenty patient-derived human tumor CRC explants were then treated with TAK-733. In total, 15 primary human tumor explants were found to be sensitive to TAK-733 (TGII ≤ 20%), including 9 primary human tumor explants that exhibited tumor regression (TGII > 100%). Explants with a BRAF/KRAS/NRAS mutant and PIK3CA wild-type genotype demonstrated increased sensitivity to TAK-733 with a median TGII of −6%. MEK-response gene signatures also correlated with responsiveness to TAK-733 in KRAS-mutant CRC.

**Conclusions:**

The MEK inhibitor TAK-733 demonstrated robust antitumor activity against CRC cell lines and patient-derived tumor explants. While the preclinical activity observed in this study was considerable, single-agent efficacy in the clinic has been limited in CRC, supporting the use of these models in an iterative manner to elucidate resistance mechanisms that can guide rational combination strategies.

## INTRODUCTION

The RAS-RAF-MEK-ERK (MAPK) pathway is a major contributor to cell growth and survival and is frequently dysregulated in numerous cancers [6, 9]. Signaling through the MAPK pathway is known to be complex with numerous downstream effector signaling pathways and can be initiated by several growth factor receptors, including the epidermal growth factor receptor (EGFR). Once the receptor is activated, it serves to activate membrane bound RAS which can then recruit RAF to the membrane. RAS thus serves as a critical link between growth factor receptors and initiation of signal transduction. RAS proteins are comprised of 4 major forms, HRAS, NRAS, and 2 forms of KRAS while RAF includes three kinase family members, ARAF, BRAF, and CRAF. The complexity of RAF activation is increased by additional non-RAS signaling activities including phosphorylation (p21 activated kinase) and dephosphorylation (protein phosphatase 2A) that are required to fully activate RAF function. RAF function is also regulated by interactions with other proteins including 14-3-3 proteins and heat shock protein 90 (Hsp90) [1]. RAF activation leads to its binding with a scaffold-like protein complex in the cytoplasm that allows it to physically locate near the vicinity of MEK1/2. MEK1 and 2 have only one known substrate, ERK [2], whereas ERK1 and ERK 2 are known to have over 160 different targets including cytosolic proteins and numerous transcription factors [3]. The RAS/RAF/MEK/ERK cascade is a central signaling pathway required for normal cellular proliferation and transformation, and MEK has been shown to be integral in the development and progression of colorectal cancer [4].

Due to the frequent aberration of this signaling cascade in malignant tissues, MEK has emerged as an attractive target in cancer. Inhibition of MEK impairs proliferation and affects a diverse array of cellular events including differentiation, apoptosis, and angiogenesis [5, 6]. MEK is a validated target in several malignancies, including non-small lung cancer and melanoma with selumetinib and trametinib respectively [7, 8]. MEK inhibitors have also shown promise in preclinical studies of KRAS/NRAS/BRAF mutant colorectal cancer, but early phase clinical trials with the MEK inhibitor selumetinib (AZD6244 hydrogen sulfate) failed to demonstrate significant improvement in progression-free survival [9, 10].

MEK1/2 (MAP2K1/K2), the canonical targets of MEK inhibitors, are dual-specificity threonine/tyrosine kinases that are integral in the activation of the RAS/RAF/MEK/ERK pathway and are often upregulated in a variety of tumor cell types. TAK-733 is a highly potent and selective novel MEK allosteric site inhibitor with an IC_50_ of 3.2 nM that selectively binds to and inhibits the activity of MEK1/2, preventing the activation of MEK1/2-dependent effector proteins and transcription factors. TAK-733 has demonstrated potent anticancer activity in several solid tumor mouse xenograft models and exhibited potent enzymatic and cell activity with an EC_50_ of 1.9 nM against ERK phosphorylation, the downstream target within the RAS/RAF/MEK/ERK pathway, in cells [11, 12].

In this study, the antitumor activity of TAK-733 was assessed against colorectal cancer cell lines and patient-derived tumor xenografts (PDX). Given the known resistance mechanisms of MEK inhibition in colorectal cancer, we hypothesized that tumors with known KRAS/NRAS or BRAF mutations that were PIK3CA wild-type would exhibit greater sensitivity to MEK inhibition [13].

## RESULTS

### Effects of TAK-733 on proliferation of colorectal cancer cell lines

**Figure 1:**
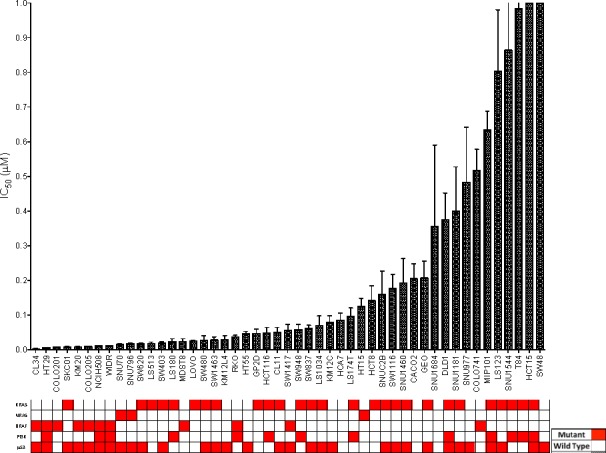
CRC cell lines exposed to TAK-733 to establish their IC50s Cell lines with an IC50 of > 0.5μM are considered to be resistant. There was a broad range of sensitivity to the agent. Mutant genes are shown in red. Eighty-two percent of sensitive cell lines were BRAF or KRAS mutant (*p* = 0.03).

Initially, a panel of 54 CRC cell lines was exposed to TAK-733 to establish the IC_50_s. As depicted in Figure 1, 54 CRC cell lines were segregated into highly sensitive (IC_50_ ≤ 0.03μM) or highly resistant (IC_50_ > 1μM). Of the 54 cell lines, 42 (77%) were classified as sensitive to TAK-733. Cell lines with a KRAS/NRAS or BRAF mutation were associated with sensitivity to TAK-733 (*p* = 0.03), and even greater sensitivity was observed in 14 of 17 CRC cell lines that were KRAS/NRAS mutant and PIK3CA wild-type.

### Effects of TAK-733 on CRC cell lines by immunoblotting

**Figure 2:**
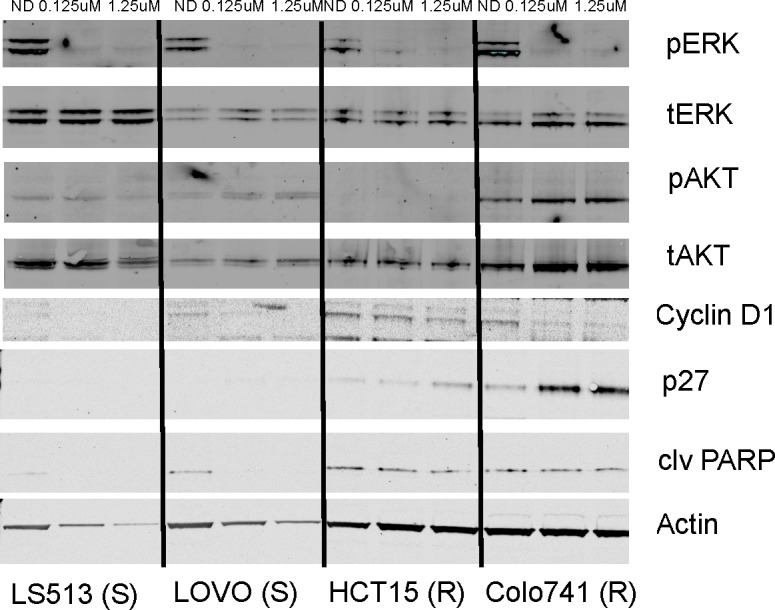
Effect of TAK-733 on downstream effectors Two sensitive and resistant CRC cell lines were exposed to TAK-733. S and R represent sensitivity or resistance to TAK-733.

The effects of TAK-733 on the modulation of downstream targets in the MAPK and PI3K pathways were analyzed in 2 sensitive and 4 resistant cell lines (Figure 2). As observed by us and others in prior studies, inhibition of p-ERK was observed in both S and R cell lines, with only one of the R cell lines (LS123) exhibiting inhibition at only the higher (1.25uM) concentration [14-16]. Interestingly, there was evidence of increased p-AKT after exposure to TAK-733 in one of the R cell lines (Colo741). Two additional TAK-733 R cell lines were evaluated, and one R cell line was found to have an increase in p-AKT (LS123) (Supplemental Figure 1). An increase in p-AKT was also observed in one of the S cell lines (LOVO).

### MEK pathway inhibition by TAK-733 in patient-derived CRC xenografts

**Figure 3:**
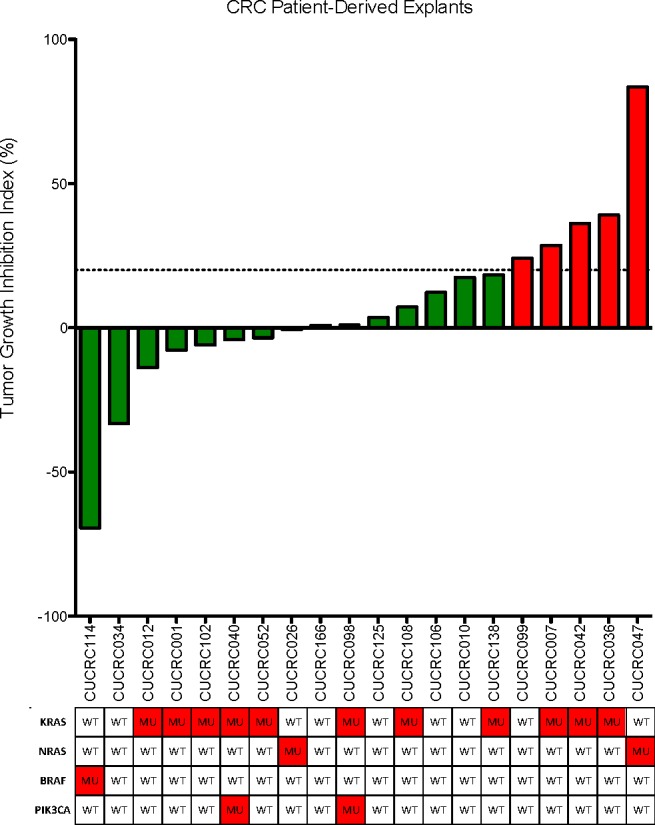
Tumor growth inhibition index (TGII) of all explants: TGII = treated over control, thus lower numbers indicate greater tumor reduction Fifteen explants were found to be sensitive to TAK-733. KRAS/BRAF mutant and PIK3CA wild-type demonstrated increased sensitivity to TAK-733.

To further investigate this agent, we conducted *in vivo* experiments in patient-derived CRC xenograft models (PDX). Based on our *in vitro* results, we assessed the ability of this molecular classifier: BRAF MUT (any PI3K), *or* KRAS/NRAS MUT and PI3K WT to predict responsiveness to TAK-733 in 20 CRC PDXs (Figure 3). To do this, we selected more clinically relevant criteria for categorizing the PDX as “responsive” or “resistant”, requiring the tumor growth inhibition index to be ≤ 20% to score a PDX as “responsive” while a TGII > 20% was classified as “resistant”. Table 1 depicts the mutational status of the PDX. Overall, there was a 75% TGII “response rate” with 15 responders using the criteria described above. There was a trend towards greater TGII in PDXs that were KRAS/BRAF mutant and PIK3CA wild-type. Notably, of the 12 KRAS/NRAS/BRAF mutant and PIK3CA wild-type tumors, 8 (67%) exhibited stable disease or partial response per TGII criteria. Interestingly, of the 8 PDXs that demonstrated tumor regression, 6 (75%) were KRAS/BRAF mutant and PIK3CA wild-type, whereas the other 2 were either all wild-type, or all mutant for RAS/RAF or PI3KCA.

**Table 1:** Mutational status of all treated PDX

PDX	KRAS	NRAS	PIK3CA	BRAF
001	MUT (G12D)	WT	WT	WT
007	MUT (G13D)	WT	MUT (3′UTR)	WT
010	WT	WT	WT	WT
012	MUT (G12V)	WT	WT	WT
026	WT	MUT	WT	WT
034	WT	WT	WT	WT
036	MUT (G12A)	WT	WT	WT
040	MUT (G12V)	WT	MUT (543)	WT
042	MUT (G13D)	WT	MUT (3′UTR)	WT
047	WT	MUT (Q61K)	WT	WT
052	MUT (G12V)	WT	WT	WT
098	MUT (G13D)	WT	MUT (E542K)	WT
099	WT	WT	WT	WT
102	MUT (G12V)	WT	WT	WT
106	WT	WT	WT	WT
108	MUT (G12C)	WT	WT	WT
114	WT	WT	WT	MUT (V600E)
125	WT	WT	WT	WT
138	MUT (G12D)	WT	WT	WT
166	WT	WT	WT	WT

### Pharmacodynamic markers of MEK pathway inhibition with TAK-733

**Figure 4:**
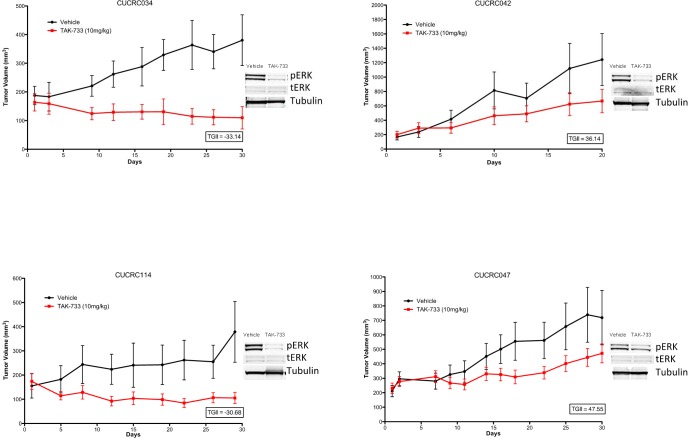
Individual growth curves of 2 sensitive and 2 resistant CRC patient-derived tumor explants (PDX) showing decreases in pERK in TAK-733 treated explants

**Figure 5:**
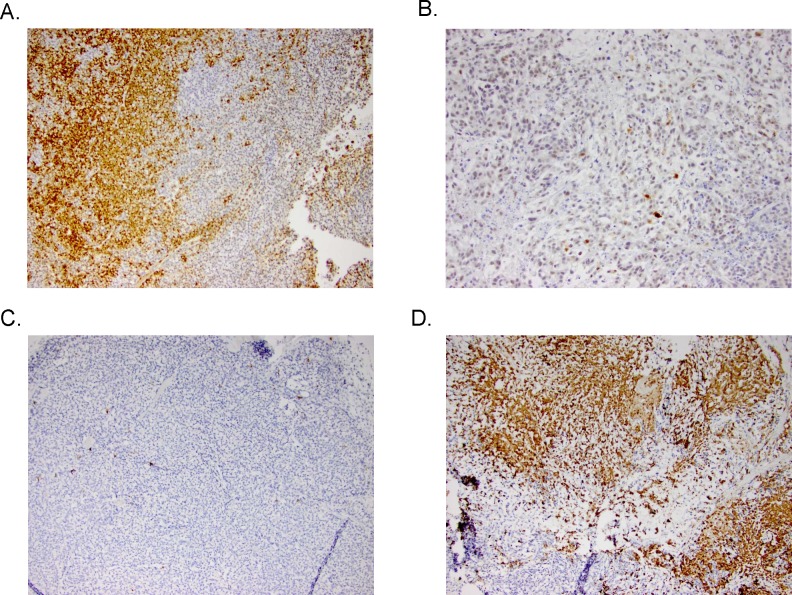
Representative IHC stains of p-ERK in A) control and B) treated PDXs (top) and caspase 3 in control and treated PDXs (bottom)

Analyses of downstream effector modulation at the end of study in 2 sensitive and 2 resistant PDX models treated with TAK-733 are depicted in Figure 4. As observed in the cell lines, suppression of p-ERK was observed in all tumors independent of responsiveness, although one could argue there was a more robust effect in the most sensitive tumor, CUCRC114, with a TGII of −67% that was accompanied by a reduction in p-AKT. The other biomarkers assessed were quite variable such as survivin, which was paradoxically decreased in the two non-responsive tumors and increased in one of the sensitive tumors, perhaps confounded by its assessment at the end of study. Next tumor samples from a responsive PDX (CUCRC 102) collected at the end of study were evaluated by immunohistochemistry (IHC). As depicted in Figure 5, after treatment with TAK-733, this PDX demonstrated the expected decrease in p-ERK with an increase in caspase 3. Similar results were observed in IHC stains performed on CUCRC102.

### Development of a MEK-sensitivity signature for KRAS mutant CRC

As KRAS mutation is a negative predictor for EGFR-based therapy for CRC patients, novel therapeutics are urgently needed for this population. Recent studies have suggested that the KRAS-mutant cancer cell lines, including CRC could be divided into two groups based on their “KRAS-dependency” [17, 18]. Based on the observation that the presence of a KRAS mutation was associated with sensitivity to a wide-range of MEK inhibitors including TAK-733 in CRC, but was insufficient for prediction alone, we reasoned that gene expression changes within this subset might enable better prediction of responsiveness to MEK inhibition. To test this, we focused on 11 KRAS mutant CRC cell lines that have been tested in our laboratory and demonstrated consistent sensitivity or resistance across four different MEK inhibitors [TAK-733, AZD6244 [19], PD-0325901 [20] and U0126 [21]] (Table 2). Using SAM analysis, we identified 201 probe sets that were differentially expressed in MEK inhibitor sensitive or resistant cell lines. To test whether these probe sets were predictive in KRAS mutant PDX models, we performed cluster analysis on the common 117 probe sets found between the cell lines and PDX models from two different platforms. From the 11 KRAS mutant PDX models treated with TAK-733, 7 and 4 models were predicted as sensitive or resistant, respectively (Table 3). The MEK signature correctly predicted 8 out of the 11 PDX models (accuracy 73%) in TAK-733 sensitivity. The sensitivity and specificity of the MEK signature to TAK-733 are 86% (6/8) and 67% (2/3), respectively. We also tested the published KRAS-dependency signature against these 11 PDX models, and this signature achieved 64% accuracy in predicting TAK-733 sensitivity. The KRAS-dependency signature has 83% and 40% for sensitivity and specificity, respectively. When we tested the MEK signature against the eight KRAS wild-type and one BRAF mutant PDX models treated with TAK-733, the prediction accuracy was only 44%, suggesting that the MEK signature is only predictive for KRAS mutant CRC (Table 4).

**Table 2:** KRAS mutant CRC cell lines sensitivity across four MEK inhibitors

CRC Cell Lines	TAK733 (This Paper)	AZD6244 (Tentler et al. MCT2010)	PD-901 (Pitts et al. PLoS ONE2014)	U0126 (Flanigan et al. CCR2013)
LOVO	S	S	S	nd
SKCO1	S	S	nd	nd
LS513	S	S	nd	S
SW403	S	S	nd	nd
LS1034	S	S	S	nd
SW620	S	S	nd	S
LS123	R	R	R	R
HCT15	nd	R	R	R
DLD1	R	nd	R	nd
GP2D	R	nd	R	nd
T84	R	nd	nd	nd

Note: nd = not determined

**Table 3:** Prediction of the MEK inhibitor sensitivity signature and KRAS-dependency signature in KRAS mutant CRC PDX models treated with TAK-733

Explants	TAK-733 Response (TGII %)	TAK-733 Response (TGII < 20% = S; TGII > 20% = R)	MEKi Signature (This Paper)	KRAS-dependency Signature (Singh et al. Cell 2012)
CUCRC012	−14	S	S	IND
CUCRC001	−8	S	S	DEP
CUCRC102	−6	S	R	DEP
CUCRC040	−4	S	S	IND
CUCRC052	−4	S	R	IND
CUCRC098	1	S	S	DEP
CUCRC108	7	S	S	DEP
CUCRC138	18	S	S	DEP
CUCRC007	29	R	S	DEP
CUCRC042	36	R	R	IND
CUCRC036	39	R	R	IND

**Table 4:** Prediction of the MEK inhibitor sensitivity signature in KRAS wildtype or BRAF mutant CRC PDX models treated with TAK-733

Explants	TAK-733 Response (TGII %)	TAK-733 Response (TGII < 20% = S; TGII > 20% = R)	MEKi Signature (This Paper)
CUCRC034	−33	S	R
CUCRC114 (BRAF mut)	−31	S	R
CUCRC026	−1	S	S
CUCRC166	1	S	R
CUCRC125	4	S	R
CUCRC106	12	S	R
CUCRC010	17	S	S
CUCRC099	24	R	R
CUCRC047	84	R	R

## DISCUSSION

TAK-733 is a highly potent and selective novel MEK allosteric site inhibitor and selectively binds to and inhibits the activity of MEK1/2. The purpose of this study was to evaluate the antitumor activity of TAK-733 in colorectal cancer cell lines and PDX models. We also developed a MEK-sensitivity signature based on four different MEK inhibitors from colorectal cancer cell lines and evaluated the predictivity of this signature in TAK-733 sensitivity in PDX models. As the results demonstrate, TAK-733 exhibited significant activity against colorectal cancer cell lines and PDX models, supporting inhibition of this pathway as a therapeutic strategy in CRC, with the caveat that better prediction is needed for single agent use, or the development of rational combinations.

Prior clinical studies with an earlier generation MEK inhibitor, selumetinib, failed to demonstrate significant improvements in progression-free survival in CRC [10]. In terms of preclinical activity, TAK-733 differs from selumetinib in its potency and ability to inhibit MEK1/2 activity with an IC_50_ for MEK1/2 of 3.6 nM compared to that of selumetinib of 14 nM [22]. In a prior study of selumetinib, roughly half of the cell lines exposed to selumetinib had IC_50_ values > 1μM, whereas in the current study, 42 of 54 CRC cell lines exhibited robust sensitivity to TAK-733, as defined by an IC_50_ ≤ 0.02 μM, with the majority being KRAS or BRAF mutant. Similar to studies of other MEK inhibitors, phospho-ERK was consistently suppressed to varying degrees and did not correlate with sensitivity. Likewise, other downstream effectors were variably impacted by TAK-733 and no clear conclusions could be made with respect to resistance mechanisms, although further studies are planned. These and other data suggest that at least in CRC, various inherent and adaptive resistance pathways exist to MEK inhibition that will require rational strategies for combination therapy [14, 15, 21, 23].

In order to provide a more clinically relevant preclinical platform for *in vivo* testing, we utilized patient-derived xenograft (PDX) models which may be better at recapitulating the tumor heterogeneity observed in patients in terms of gene-expression patterns, mutational status, and tumor architecture [24]. Additionally, we utilized more stringent response criteria to TAK-733 by setting a cutoff of TGII < 20, similar to RECIST measurements utilized in the clinical trial setting [25]. In our study, TAK-733 demonstrated substantial activity with 9 of 20 PDXs exhibiting tumor regression. This is unusual for a MEK inhibitor in CRC, and of published preclinical studies of selumetinib and trametinib, we could only find 3 models where regression was induced as a single agent, and this was largely in cell line xenograft models [14, 26-28]. Furthermore, there was a trend towards tumors displaying regression in KRAS/BRAF mutant and PIK3CA wild-type status, suggesting a potential hypothesis that can be tested in future preclinical studies of TAK-733. Similar to what was observed in cell lines, downregulation of p-ERK was consistently observed regardless of response and it was difficult to ascertain the contribution of other potential resistance pathways at the end of study, although robust caspase 3 induction was observed in a model with significant regression. While prior studies of MEK inhibition in CRC and melanoma have indicated resistance through PI3K pathway activation, our results were not consistent across multiple models, and may in fact, reflect the fact that at least in the case of CRC, combinations of PI3K and MEK inhibitors have not been particularly active in the clinic [29, 30]. As has been reported by us and others, resistance to MEK inhibition in CRC is multifactorial and related to secondary mutation events, feedback loops, or compensatory pathway activation, all of which require improved detection methodology so that combination therapy can be individualized [15, 31].

Not surprisingly, there was significant activity of TAK-733 against KRAS mutant CRC, due to constitutively active MEK and ERK phosphorylation in this subset. Structural and functional analyses have indicated that MEK inhibitors with superior anti-tumor activity in KRAS-driven tumors form a strong hydrogen-bond interaction with the backbone amide of S212 in MEK that is critical for blocking MEK feedback phosphorylation by wild-type RAF [32]. The pyridine oxygen within the structure of TAK-733 is able to form a hydrogen bond as described above, and this interaction coupled with the potent inhibition of phosphorylated MEK may explain the anti-tumor activity observed in KRAS and BRAF mutant models, whereas activity in the non-mutant models continues to be a mystery that warrants further study.

From the MEK inhibitor signature, *KRAS* and *SPRY2* were among the highly expressed genes in the sensitive group. Both genes are regulators of the MAPK signaling pathway and thus the sensitive lines are “dependent” on this pathway. Among the genes highly expressed in the MEK signature that predict resistance to this class of inhibitors are *FZD2*, a biomarker that we previously described as modulating resistant to AZD6244 *via* non-canonical Wnt pathway [15, 19]. We further demonstrated the combination of AZD6244 and Cyclosporin A (calcineurin inhibitor) is synergistic in KRAS mutant CRC PDXs [15]. This combination has been translated into a Phase I/II clinical trial (ClinicalTrials.gov ID: NCT02188264) at our institute. Another highly expressed gene in the MEK signature in the resistant group is anti-apoptotic gene *BCL2L12*, a BCL2-family member. A recent synthetic lethality screen of MEK inhibitor (AZD6244) in KRAS mutant cancer identifies the anti-apoptotic gene BCL-XL as the top hit. The combination of ABT-263 (navitoclax, a chemical inhibitor of the BCL2 family) and a MEK inhibitor shows synergistic effects in KRAS mutant CRC xenografts [23], and this combination is currently being tested in a Phase I/II clinical trial (ClinicalTrials.gov ID: NCT02079740). Taken together, the MEK inhibitor sensitivity signature is biologically relevant and provides a list of candidate resistant genes for future combination studies with MEK inhibitors.

In summary, TAK-733 is a potent and selective MEK allosteric site inhibitor demonstrating significant activity against CRC cell lines and PDXs with KRAS and BRAF mutations. In particular, some CRC PDX models exhibited significant tumor regression, particularly those harboring mutations in KRAS and BRAF with no mutation in PIK3CA. This activity in CRC provides a rationale for further clinical study in patients with advanced CRC with a potential patient-selective biomarker strategy focusing on KRAS and BRAF mutant, PIK3CA wild-type tumors. Further studies will need to focus on elucidating mechanisms of resistance to TAK-733 and strategies to overcome resistance pathways with novel combination therapies.

## MATERIALS AND METHODS

### Cell culture and proliferation analysis

All human colon cancer cells were grown in RPMI medium supplemented with 10% fetal bovine serum (FBS), 1% nonessential amino acids, and 1% penicillin/streptomycin and were maintained at 37°C in an incubator under an atmosphere containing 5% CO_2_. The cells were routinely screened for the presence of mycoplasma (MycoAlert; Cambrex Bio Science) and were exposed to TAK-733 when they reached approximately 70% confluence. All cell lines were tested and authenticated by the University of Colorado Cancer Center DNA Sequencing and Analysis Core. DNA from CRC cell lines was analyzed using the Profiler Plus Kit (Applied Biosystems).

The antiproliferative effects of TAK-733 against CRC cell lines were determined using the sulforhodamine B (SRB) method. Briefly, cells in logarithmic growth phase were transferred to 96-well flat-bottomed plates with lids. Cell suspensions (100 μL) containing 3,000 to 5,000 viable cells were plated into each well and incubated overnight before exposure with increasing concentrations of TAK-733 for 72 hours. After treatment, medium was removed and the cells were fixed with cold 10% TCA for 30 minutes at 4°C. The cells were then washed with water and stained with 0.4% SRB (Fisher Scientific) for 30 minutes at room temperature and washed again with 1% acetic acid followed by stain solubilization with 10 mmol/L of Tris at room temperature. The plate was then read on a 96-well plate reader (Biotek Synergy 2) set at an absorbance wavelength of 565 nm. Cell proliferation curves were derived from the raw absorbance data and expressed as the percentage of vehicle-treated controls.

### Immunoblotting analysis

Cells were initially plated into 6-well plates and cultured in RPMI with 10% FBS for 24 hours. All cells were then cultured in serum-free RPMI medium for 16 hours to lower the basal levels of ERK and AKT phosphorylation. The cells were treated with vehicle or TAK-733 for 2 hours and then exposed to 10% FBS or serum-free media for 10 minutes. After treatment, the cells were immediately disrupted in RIPA lysis buffer containing protease and phosphatase inhibitors (50 mmol/L of Tris-HCL, pH 7.4, 150 mmol/L of NaCl, 1 mmol/L of PMSF, 1 mmol/L EDTA, 5 μg/mL of aprotonin, 5 μg/mL of leupeptin, 1% Triton X-100, 1% sodium deoxycholate, and 0.1% sodium dodecyl sulfate). Forty micrograms of total protein was loaded onto a 10% polyacrylamide gel, electrophoresed, and then transferred to nitrocellulose using the G2 Fast Blotter (Pierce). Membranes were blocked for 1 hour in blocking buffer [0.1% casein solution in 0.2× phosphate buffered saline (PBS)] and were then incubated overnight at 4°C in blocking buffer plus 0.1% Tween-20 with the primary antibodies (Cell Signaling). Blots were then washed 3 × 10 minutes in 1× TBS containing 0.1% Tween-20 and incubated with the appropriate secondary goat anti-rabbit and goat anti-mouse IgG (H + L) DyLight™ conjugated antibodies at 1:15,000 (Cell Signaling) for 1 hour at room temperature. Following 3 × 10 minutes of washes, the blots were developed using the Odyssey Infrared Imaging System (LI-COR Biosciences). Immunoblot experiments were done in triplicate for each antibody and representative blots are depicted.

### Patient-derived tumor explant models

PDX establishment and treatment protocols were conducted under previously described procedures [33, 34]. Briefly, surgical specimens from patients undergoing either removal of a primary CRC or metastatic tumor at the University of Colorado Hospital were reimplanted s.c. into five mice for each patient. Tumor samples were then passaged into subsequent generations of mice for drug studies. Briefly, tumors were allowed to grow to a size of 1,000 to 1,500 mm^3^ (F1) at which point they were harvested, divided, and transplanted to an additional five mice (F2) to maintain the tumor bank. After a subsequent growth passage, tumors were excised, transplanted onto both flanks of nude mice, and expanded into cohorts of ≥25 mice for treatment. Tumors from this cohort were allowed to grow until reaching approximately 150 to 300 mm^3^, at which time they were equally distributed by size into the two treatment groups: control and TAK-733 treated. Because of the variability in take rates of the human patient explant material, enough mice were designated into each group based on the number of overall tumors (*n* = at least 12 tumors per group). Mice were treated for at least 28 days with either vehicle control (0.5% methylcellulose) or TAK-733 (1 mg/kg) once daily by oral gavage. Tumor growth inhibition index was calculated from average volume of the treated (*V*_t_) and vehicle control (*V*_vc_) groups, with the equation: TGII = 100 × (*V*_t final_ -*V*_t initial_)/(*V*_vc final_ -*V*_vc intial_).

### Gene expression analysis and the development of a MEK sensitivity signature

Raw microarray gene expression data for the KRAS mutant CRC cell lines was obtained from the Cancer Cell Lines Encyclopedia (GSE36133). These samples were profiled by the Affymetrix HG-U133 Plus 2 arrays. Gene expression profiles were normalized by RMA and extracted using Affymetrix Power Tools (APT). Significant Analysis of Microarrays (SAM) (PMID: 11309499) was performed using R, with 500 permutations. Probe sets that passed FDR < 0.25 were selected as significantly differentially expressed in MEK sensitive and resistant KRAS mutant CRC cell lines. Raw microarray gene expression data for the CRC PDX models were profiled by the Affymetrix HuGene 1.0 arrays. Gene expression profiles were normalized by RMA and extracted using APT. Probe sets were matched between platforms using the BEST_MATCH probe sets provided by Affymetrix. Probe sets that matched between two platforms were Z-normalized independently and merged into single gene expression profiles for cluster analysis. We used Spearman's rank correlation with Average linkage analysis in Cluster 3.0 (PMID: 14871861) and visualized in Java TreeView for the cluster analysis.

## SUPPLEMENTARY MATERIAL FIGURE


