# Supplementary material for: Antitumor activity of a potent MEK inhibitor, TAK-733, against colorectal cancer cell lines and patient derived xenografts
Source: Oncotarget. 2015 Oct 1;6(33):34561–72. doi: 10.18632/oncotarget.5949 (PMC4741473; doi:10.18632/oncotarget.5949)
Supplement: Supplementary file 1 [file oncotarget-06-34561-s001.pdf]

Antitumor activity of a potent MEK inhibitor, TAK-733, against colorectal cancer cell lines and patient derived xenografts

Supplementary Material

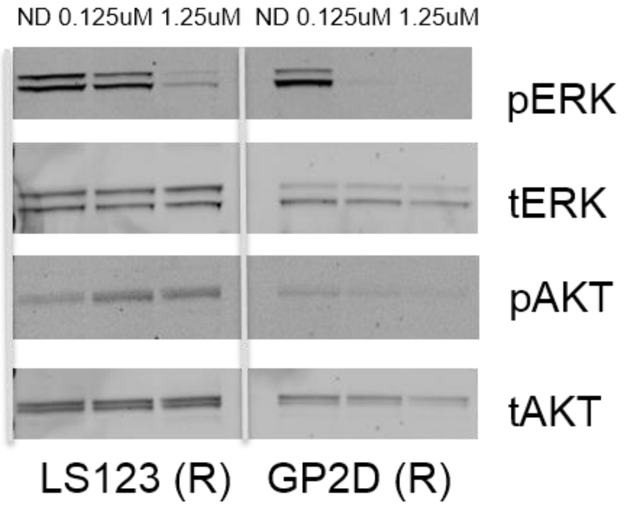

Supplemental Figure 1. Effect of TAK-733 was evaluated on downstream effectors in two TAK-733-resistant cell lines with one cell line (LS123) showing an increase in p-AKT.
